# Accounting for variation in temperature and oxygen availability when quantifying marine ecosystem metabolism

**DOI:** 10.1038/s41598-021-04685-8

**Published:** 2022-01-17

**Authors:** Matthew E. S. Bracken, Luke P. Miller, Sarah E. Mastroni, Stephany M. Lira, Cascade J. B. Sorte

**Affiliations:** 1 Department of Ecology and Evolutionary Biology, University of California - Irvine, 321 Steinhaus Hall, Irvine, CA 92697-2525 USA; 2 Department of Biology, San Diego State University, 5500 Campanile Drive, San Diego, CA 92182 USA; 3 Present Address: Coastal Science and Policy Program, University of California - Santa Cruz, 115 McAllister Way, Santa Cruz, CA 95060 USA

**Keywords:** Ecosystem ecology, Marine biology

## Abstract

It is critical to understand how human modifications of Earth’s ecosystems are influencing ecosystem functioning, including net and gross community production (*NCP* and *GCP*, respectively) and community respiration (*CR*). These responses are often estimated by measuring oxygen production in the light (*NCP*) and consumption in the dark (*CR*), which can then be combined to estimate *GCP*. However, the method used to create “dark” conditions—either experimental darkening during the day or taking measurements at night—could result in different estimates of respiration and production, potentially affecting our ability to make integrative predictions. We tested this possibility by measuring oxygen concentrations under daytime ambient light conditions, in darkened tide pools during the day, and during nighttime low tides. We made measurements every 1–3 months over one year in southeastern Alaska. Daytime respiration rates were substantially higher than those measured at night, associated with higher temperature and oxygen levels during the day and leading to major differences in estimates of *GCP* calculated using daytime versus nighttime measurements. Our results highlight the potential importance of measuring respiration rates during both day and night to account for effects of temperature and oxygen—especially in shallow-water, constrained systems—with implications for understanding the impacts of global change on ecosystem metabolism.

## Introduction

It is essential to understand impacts of climate change on marine ecosystem functioning, especially given observed and accelerating increases in ocean temperatures^1–3^, acidification^1,4–6^, and hypoxia^1,6,7^. A key set of ecosystem-level responses—related to carbon fixation and availability in marine systems—is changes in productivity^1,8^ and respiration^9^, including net and gross community production^10,11^. Accordingly, community production and respiration have been measured for over a century; light and dark bottles were first used to estimate marine community production in 1916^12^. Since then, a variety of methods, some of which produce substantially differing results, have been used to quantify and calculate community production rates^13–15^. Given the importance of these measurements for understanding ecosystem responses to global change^8,9^, we evaluate the effectiveness of two of the most commonly used methods for estimating productivity—day-night measurements versus daytime light–dark incubations—highlighting differences between them and exploring the mechanisms underlying those differences.

These methods for measuring ecosystem metabolism are based on the long-understood fact that respiration causes CO_2_ release and O_2_ consumption in both the dark and the light, whereas photosynthesis drives carbon fixation and oxygen generation only in the light^12,16^. Key metrics of ecosystem metabolism (sensu Beyers^17^) are gross community production (*GCP*), which is the rate of photosynthetic carbon fixation (or oxygen production) in the community being measured; community respiration (*CR*), which is the rate of carbon release or oxygen consumption, and net community production (*NCP*), which is the rate of observed carbon fixation or oxygen accumulation in the light, including the effects of respiration on carbon or oxygen^18,19^. Operationally, *GCP* is derived by adding *CR* (i.e., respiration) to *NCP* (i.e., gross production *minus* respiration^19,20^).

However, methods used to measure and calculate ecosystem metabolism—in-situ measurements and closed containers— differ in their underlying assumptions and, therefore, potential accuracy^21^. In-situ changes in dissolved oxygen in a body of water over time—including both night and day—are associated with photosynthesis and respiration and can be used to estimate community production and respiration rates^16,22–25^. These “free-water” estimates (sensu Odum and Hoskin^23^) are contrasted with measurements in closed containers, where a trapped assemblage of organisms is exposed to light and dark conditions, and measured responses are changes in oxygen concentrations^12,26^ or uptake of radiocarbon^27,28^. Typically, incubations of closed containers are made for 24 h (e.g., dawn-to-dawn) to encompass both night and daytime, and some containers are transparent (measuring both photosynthesis and respiration), whereas others are darkened (measuring only respiration)^15,23^.

Issues and artefacts have been identified with both free-water and closed-container methods. Closed containers have been criticized for a variety of reasons: nutrients cannot be replenished so nutrient depletion over time can limit productivity, biofilms can develop on the bottle surfaces, incubation conditions (e.g., shipboard) may not accurately reflect natural conditions (e.g., temperature, light), and bottles cannot effectively capture benthic processes^23,24,26^. At the same time, “free-water” methods assume that nighttime and daytime-dark conditions are equivalent, that advection of water masses is negligible or can be accounted for, and that diffusion from the atmosphere can be measured or modeled^22,23,29,30^. The effectiveness of each method is likely contingent on the system and context.

In-situ measurements clearly have some advantages in capturing whole-system dynamics, especially when the system includes both pelagic and benthic components. However, when respiration is only measured at night, as in the free-water method, the assumption is that nighttime and daytime respiration are the same^22,31^, and estimates of gross primary production depend on this assumption^32,33^. Similarly, when respiration is only measured using darkened containers during the day, the assumption is that nighttime conditions can be effectively replicated during the day, provided that light does not affect respiration rates^34^. Here, we take both nighttime and daytime-darkened measurements to test these assumptions. In particular, the equivalence of daytime and nighttime respiration rates remains largely untested (but see Mantikci et al.^34^), especially in shallow-water systems where conditions (e.g., temperature, oxygen availability) can vary substantially between night and day^35–38^.

We conducted measurements of oxygen changes in daytime “ambient” light conditions, in darkened conditions during the day, and at night to evaluate the assumption that nighttime respiration rates can be used to infer daytime respiration and gross primary production. Measurements were made in tide pools—depressions in rocky reefs that remain filled with water when the tide recedes—on a shoreline in southeastern Alaska, USA. When isolated from the ocean, tide pools offer an ideal compromise between container and “free-water” methods, as it is straightforward to measure oxygen fluxes during both daytime and nighttime low tides^16^, and pools can be covered by opaque sheets during the daytime to evaluate respiration rates^19,39–41^. We used a series of measurements to evaluate the potential for methodology—especially Dark (i.e., experimentally darkened during the day) *vs*. Night incubations—to affect estimates of production and respiration. Because of the importance of irradiance levels (light intensities) in determining productivity rates^42^, the primary factor that we varied in our methods was light availability, either by covering pools with opaque plastic sheets during the day or by measuring oxygen fluxes at night. Given that community composition, especially the abundances of algae and mobile invertebrates, changes seasonally in these tide pools (Sorte et al., unpublished data), we predicted that our estimates of production (*NCP* and *GCP*) and respiration (*CR*) would vary across the year. Our comparison of methods for estimating ecosystem metabolism provides an explicit test of the assumption of “constant community respiration day and night”^22^.

## Results

### Production and respiration

**Figure 1:**
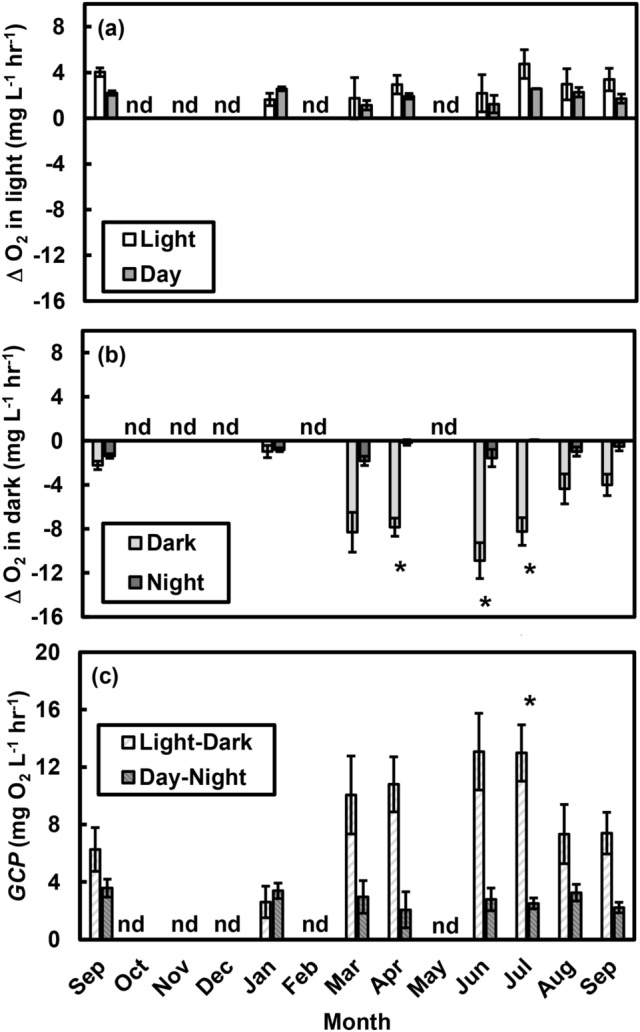
Effects of methodology on measured changes in oxygen (O_2_) concentrations and gross community production (*GCP*) in tide pools. (**a**) Measurements made in the light (estimates of net community production) generally indicated net production of O_2_, were similar regardless of method (“Light” vs. “Day”), and did not change over time. (**b**) Measurements in the dark (estimates of community respiration) typically indicated net consumption of O_2_, differed substantially depending on method (“Dark” vs. “Night”), and changed over time. **(c)** Measurements of *GCP* made consecutively during daytime (Light–Dark) resulted in higher estimates of *GCP* than those made during day and night (Day-Night). Overall, *GCP* varied with time, and the difference between methods changed with time. Values are means ± s.e., and “nd” marks months when “no data” were collected. Asterisks (*) indicate differences between methods (*p* < 0.05).

Repeated-measures analyses (see “Methods” for details) indicated that measurements conducted in the light (on different days associated with “Day” and “Light” measurements made during “Day-Night” and “Light–Dark” sampling, respectively) showed that oxygen fluxes measured using the two methods were similar across dates (*F*_1,4_ = 4.7, *p* = 0.096) and that net community production did not change seasonally (by Date; *F*_7,28_ = 1.2, *p* = 0.330; Fig. 1a). In contrast, rates of oxygen consumption in the dark were much greater when samples were collected during daytime “Dark” incubations than during nighttime “Night” sampling (*F*_1,4_ = 139.1, *p* < 0.001), and they changed substantially over time (*F*_7,28_ = 8.5, *p* < 0.001; Fig. 1b). Specifically, seasonal changes in community respiration were more pronounced in daytime “Dark” samples than in those collected at “Night” (‘Method × Date’ interaction, *F*_7,28_ = 8.5, *p* < 0.001). Because volume was closely correlated with area in these tide pools (*r*^2^ = 0.97), we observed similar patterns when measurements were expressed on a per-area basis (i.e., mg O_2_ m^−2^ h^−1^; Supplementary Fig. S1a, b).

Because of these differences in respiration between sampling methods, gross community production (*GCP*)—calculated by adding the rate of oxygen consumption in the dark to the rate of oxygen production in the light—was higher when using the “Light–Dark” method than when using the “Day–Night” method (*F*_1,4_ = 67.3, *p* < 0.001; Fig. 1c). There was seasonality in *GCP* (*F*_7,28_ = 9.0, *p* < 0.001), and seasonal variation depended on the method used (‘Method × Date’ interaction, *F*_7,28_ = 8.5, *p* < 0.001). As with the measured changes in oxygen concentrations, we observed similar patterns for *GCP* when values were quantified on a per-area basis (Supplementary Fig. S1c).

### Factors associated with variation in production and respiration

After accounting for seasonality (i.e., ‘Date’ was included in the model, *F*_7,28_ = 1.0, *p* = 0.469), there was no effect of initial light intensity (*F*_1,60_ = 0.2, *p* = 0.648) or temperature (*F*_1,60_ = 1.4, *p* = 0.228) on net community production (*NCP*, the rate of O_2_ accumulation in the light). *NCP* was influenced by the initial dissolved oxygen concentration, declining at higher O_2_ concentrations (*F*_1,60_ = 4.7, *p* = 0.033).

**Figure 2:**
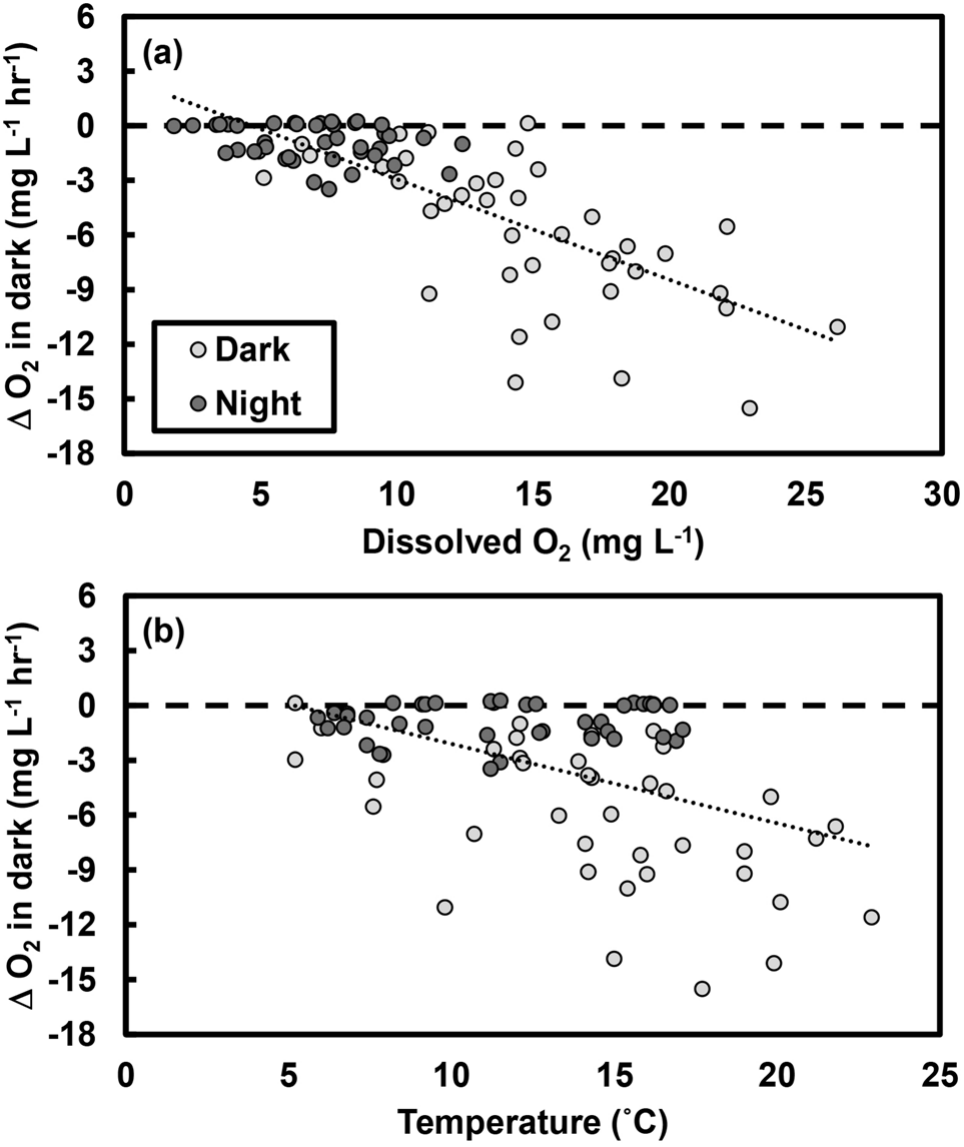
Oxygen (O_2_) consumption in the dark increased with higher levels of both (**a**) dissolved O_2_ and (**b**) temperatures, leading to greater declines in O_2_ concentrations over time.

The rate of oxygen consumption in the dark (i.e., community respiration, *CR*) was influenced by the initial oxygen concentration (Fig. 2a) and the temperature (Fig. 2b). After accounting for seasonality (i.e., ‘Date’ was included in the model, *F*_7,28_ = 4.8, *p* = 0.001), increases in both O_2_ (*F*_1,58_ = 15.4, *p* < 0.001) and temperature (*F*_1,58_ = 22.9, *p* < 0.001) were associated with greater rates of O_2_ consumption. Rates of *CR* were also higher in tide pools containing higher densities of littorine snails (*F*_1,38_ = 14.1, *p* < 0.001; Supplementary Fig. S2), which were the most abundant mobile invertebrates at our study location.

After accounting for seasonality (Date: *F*_7,25_ = 1.6, *p* = 0.180), rates of gross community production (*GCP*) were enhanced at higher temperatures (*F*_1,25_ = 6.6, *p* = 0.016) but were unrelated to irradiance (*F*_1,25_ = 1.9, *p* = 0.183) or dissolved O_2_ levels (*F*_1,25_ < 0.1, *p* = 0.861). Rates of *GCP* were also higher in pools containing higher algal cover (*F*_1,38_ = 5.1, *p* = 0.029; Supplementary Fig. S2).

**Figure 3:**
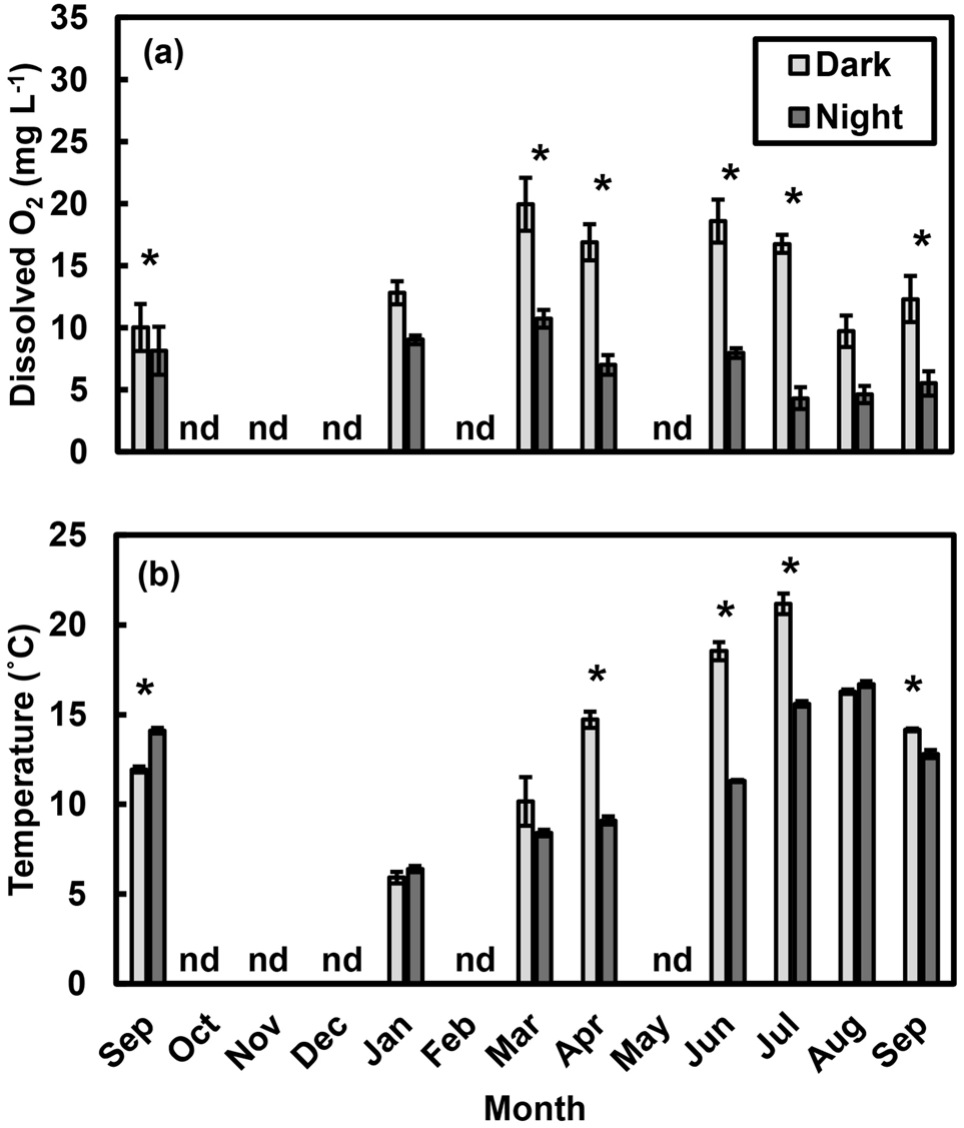
Dissolved oxygen (O_2_) levels and temperatures associated with different methods of quantifying changes in O_2_ concentrations in the dark. Both (**a**) O_2_ levels and (**b**) temperatures were higher when tide pools were covered during the day (“Dark”) than during nighttime (“Night”) sampling. Values are means ± s.e., and “nd” indicates months when “no data” were collected. Asterisks (*) indicate differences between methods (*p* < 0.05).

Initial O_2_ and temperature values were both higher during daytime “Dark” than during “Night” sampling (O_2_: *F*_1,4_ = 174.4, *p* < 0.001, Fig. 3a; temp: *F*_1,4_ = 134.7, *p* < 0.001, Fig. 3b) and were both higher in spring and summer than during fall and winter months (O_2_: *F*_7,28_ = 10.7, *p* < 0.001; temp: *F*_7,28_ = 188.8, *p* < 0.001). Furthermore, the difference between methods depended on the month (‘Method × Date’ interaction; O_2_: *F*_7,28_ = 4.4, *p* = 0.002; temp: *F*_7,28_ = 36.4, *p* < 0.001). Dissolved O_2_ and temperature did not differ between daytime “Dark” and “Night” sampling in January, when O_2_ and temperature measurements were similar during day and night, but there was divergence during late spring and early summer (April–July). After accounting for initial temperatures and O_2_ concentrations (i.e., they were included as factors in the model), there was no difference between methods (Method: *F*_1,4_ = 0.1, *p* = 0.746; Supplementary Fig. S3), and the effect of methodology on community respiration did not change over time (‘Method × Date’ interaction: *F*_7,28_ = 1.3, *p* = 0.230).

### Integrated estimates of production and respiration

**Figure 4:**
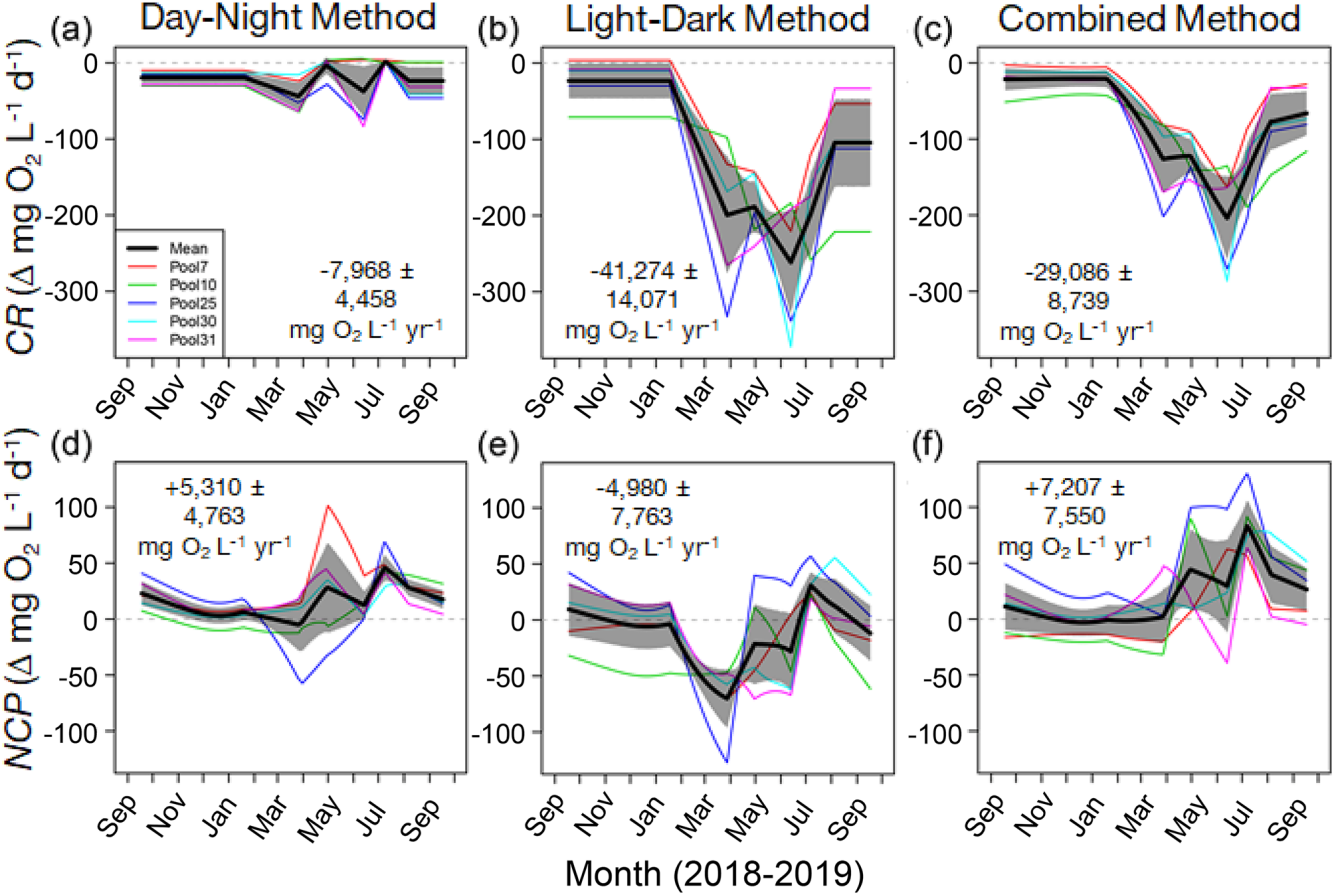
Integrated daily (mg O_2_ L^−1^ d^−1^) and annual (mg O_2_ L^−1^ year^−1^) estimates of community respiration (*CR*) and net community production (*NCP*; mg O_2_ L^−1^ day^−1^). Upper panels show estimates of *CR* and lower panels show estimates of *NCP* using (**a**, **d**) the Day–Night method, (**b**, **e**) the Light–Dark method, and (**c**, **f**) the combined method (Light–Dark during day and Day–Night during night). Black lines represent the means, and shaded polygons represent bootstrapped 95% CIs, with individual pools represented by colored lines. Integrated annual estimates are means ± bootstrapped 95% CIs.

Using “Night” values to estimate O_2_ consumption rates (Fig. 4a) resulted in considerably reduced (less negative) estimates for *CR* relative to the other two methods (Fig. 4b, c), which used daytime “Dark” measurements for one or both estimates of *CR*. Overall, using only Day–Night values resulted in annual estimates of *CR* (mg O_2_ L^-1^ yr^-1^) an order of magnitude lower than the other methods (Fig. 4a-c). Additionally, using daytime (Light–Dark) “Dark” values to estimate nighttime *CR* rates (Fig. 4b) resulted in estimates of daily O_2_ consumption that were qualitatively more rapid (i.e., more negative, indicating O_2_ consumption) than those that combined “Dark” and “Night” measurements (Fig. 4c). Whereas there was overlap between integrated annual *CR* values estimated using Light–Dark and combined methods, the Light–Dark method estimated higher O_2_ consumption when applying daytime-derived respiration to the overnight periods as well (Fig. 4b,c).

Using only “Dark” values from Light–Dark sampling (Fig. 4e) resulted in much lower rates of 24-h *NCP* (mg O_2_ L^−1^ day^−1^) than the other two methods, including a period from January to May where estimates of production were less than zero (Fig. 4d,f). Using exclusively the Light–Dark method to estimate annual *NCP* (mg O_2_ L^−1^ year^−1^) suggested that production did not exceed respiration, as average values were negative, although the 95% confidence interval spanned zero (Fig. 4e). Both the Day–Night method and the combined method indicated positive *NCP* values, but the estimate using only Day–Night sampling was lower (Fig. 4d).

**Figure 5:**
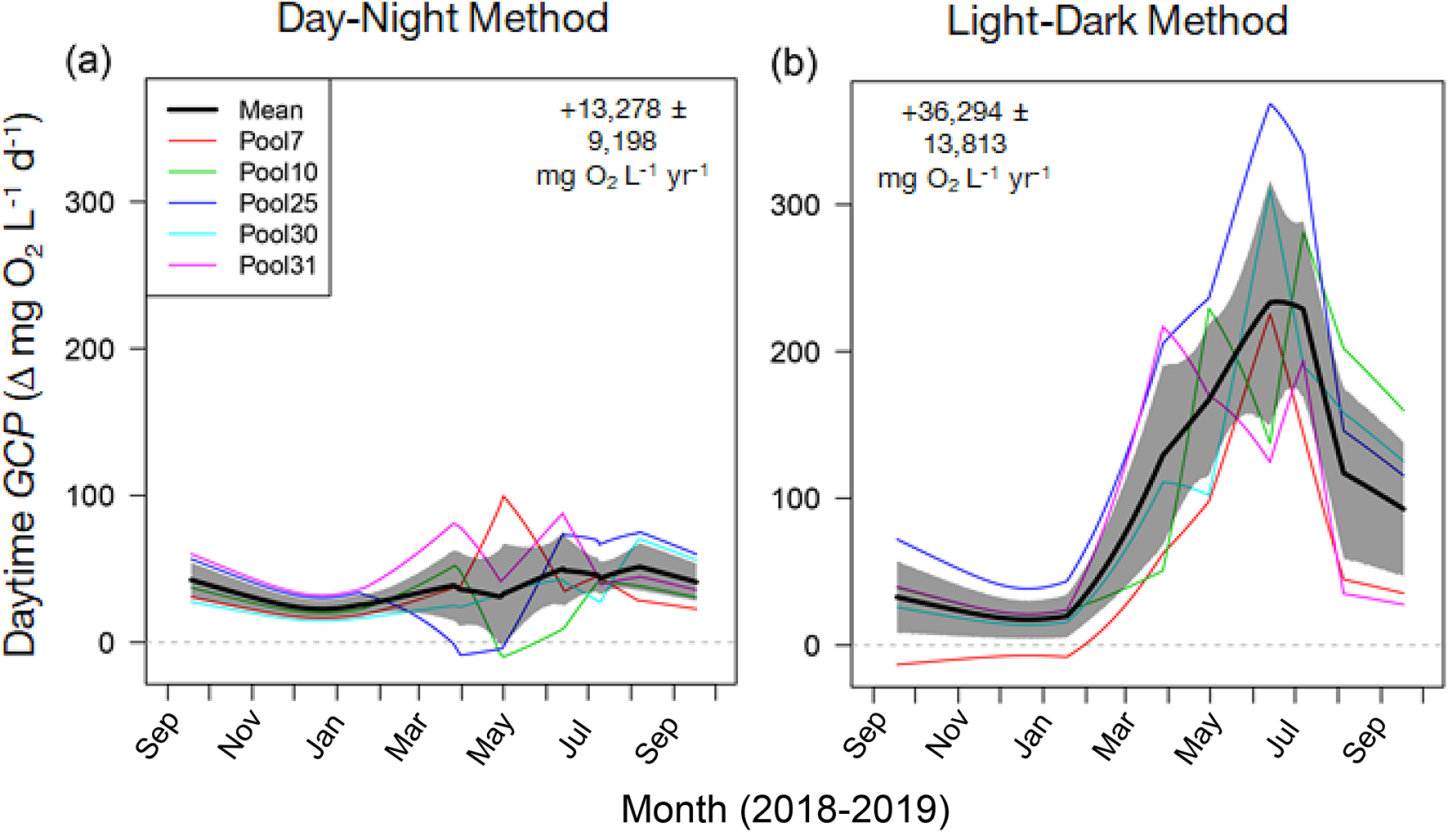
Integrated daily (mg O_2_ L^−1^ d^−1^) and annual (mg O_2_ L^−1^ year^−1^) estimates of gross community production (*GCP*) during daylight hours using the (**a**) Day–Night method and (**b**) the Light–Dark method. Black lines are the means, shaded polygons represent bootstrapped 95% CIs, and colored lines represent values from individual tide pools. Integrated annual estimates are means ± bootstrapped 95% CIs.

Using the Day–Night method to estimate daily *GCP* for daylight hours (mg O_2_ L^−1^ day^−1^; Fig. 5a) resulted in values that were considerably lower than those obtained using the Light–Dark method, especially during the summer (Fig. 5b). Similarly, annual *GCP* estimates (mg O_2_ L^−1^ year^−1^) based on the Day–Night method were substantially lower than those based on the Light–Dark method (Fig. 5).

## Discussion

Our results highlight the importance of accounting for the effects of temperature and oxygen in estimating ecosystem metabolism. The most important factor influencing net community production was O_2_ concentration (*NCP* declined as tide pool O_2_ concentrations increased, though this could also reflect reduced concentration gradients as tide pool O_2_ levels increased), and the most important factor influencing gross community production was temperature (*GCP* increased at higher temperatures). Despite our efforts to minimize the potential for high O_2_ levels to influence respiration during daytime “Dark” measurements, higher O_2_ concentrations during the day (Fig. 3a) likely reflected higher O_2_ production, potentially resulting in higher photosynthate enrichment and O_2_ consumption during those measurements. Similarly, estimates of *CR* and, consequently, *GCP* were strongly dependent on whether dark measurements were made during the day (“Dark”) or at night, suggesting that the ideal method depends on which aspects of ecosystem metabolism researchers aim to quantify. Collectively, our results indicate that a key assumption of “free-water” estimates of ecosystem metabolism—that respiration rates measured at night are equivalent to those during the day^31^—does not hold in this system. We observed substantially higher rates of O_2_ consumption (community respiration, *CR*) during daytime “Dark” incubations than during nighttime “Night” incubations (Fig. 1b). This was not entirely unexpected, as Odum noted that earlier researchers^29^ had found substantial variation in respiration rates in dark bottles between day and night^22^. However, Odum then dismissed this complication and assumed constant community respiration^22^. The daytime *CR* rates we observed were higher than those measured at night, but they were consistent with other measurements from temperate rocky shores. For example, on an areal basis, our measurements (73–372 mg O_2_ m^−2^ h^−1^; Supplementary Fig. S1b) were similar to those recorded in a series of trials on the coast of Norway (133–511 mg O_2_ m^−2^ h^−1^)^43,44^.

We found that community respiration rates were closely associated with temperature and O_2_ concentrations: O_2_ consumption increased with increasing temperatures and initial O_2_ levels (Fig. 2), and diel variation in temperature and O_2_ explained the difference between “Dark” and “Night” incubations. In fact, when O_2_ and temperature were taken into account (i.e., included in the statistical model), the differences between respiration rates measured in “Dark” and “Night” incubations were no longer evident (Supplementary Fig. S3). Given substantial diel variation in temperatures and O_2_ levels recorded in a variety of shallow marine and freshwater systems^35–38^, we suggest that the assumption of “constant community respiration day and night”^22^ should be reevaluated in some instances. To assess this possibility, we recommend measuring temperature and O_2_ levels to assess differences between day and night conditions. And whereas we did not find an effect of irradiance on respiration rates, we cannot discount the potential for light-enhanced dark respiration^45^, and we suggest measuring irradiance levels.

Daytime “Dark” community respiration rates were substantially higher than those measured at night (Figs. 1b, 4a–c), leading to correspondingly higher estimates of hourly (Fig. 1c) and integrated (daily and annual) gross community production (Fig. 5). For estimating daily and annual rates of community metabolism, we advocate a “combined” method that uses day and night measurements to calculate net community production (*NCP*; Fig. 4d,f) and that uses daytime “Dark” respiration rates during daytime hours and “Night” respiration rates during nighttime hours to estimate community respiration (*CR*; Fig. 4c). If daytime and nighttime conditions (e.g., temperature, O_2_) differ substantially, estimates of daytime gross community production (*GCP*) should be based on daytime *CR* measurements whenever possible. We predict that daily estimates of *GCP* are underestimated by up to an order of magnitude and annual estimates are almost three times lower when “Night” *CR* measurements are used (Fig. 5).

One important potential caveat to our suggestion and interpretation is the possibility for light-enhanced respiration. Higher rates of photosynthesis—associated with higher irradiance levels—are associated with higher respiration rates^34,45,46^, leading to higher respiration rates measured during the day in producer-dominated communities^47^. While this is a possible explanation for the observed differences between daytime and nighttime respiration, the fact that *GCP* was related to temperature, but not to irradiance levels, suggests that temperature was a primary factor. And when tide pool temperatures and O_2_ concentrations were taken into account in our statistical analysis of factors influencing *CR*, differences between daytime and nighttime *CR* measurements were no longer apparent (Supplementary Fig. S3). Animal respiration—and the factors that influence it—was also an important aspect of these diverse, natural communities, as highlighted by the relationship between *CR* and abundances of littorine snails. Whereas light-enhanced respiration is clearly important in many systems^34,45–47^, these findings suggest that it does not play a major role in these tide pools relative to other drivers of ecosystem metabolism.

Measurements of community production and respiration are essential for quantifying whole-system responses to anthropogenic changes in environmental conditions^48^. Both O_2_ and temperature—which we have identified as strong drivers of ecosystem metabolism (Fig. 2)—are changing at both local and global scales due to climate warming and climate-mediated reductions in O_2_ availability^1,6,7,49^. We highlight important mechanisms—temperature and O_2_ effects on community respiration (*CR*) rates (Fig. 2)—by which these changing environmental conditions can modify ecosystem functioning. These effects are, therefore, not only methodological considerations, but they also provide insights into impacts of global change on ecosystems. We found that *CR* responded strongly to changes in temperature and O_2_ levels, which is consistent with a greater response of respiration than production to ocean warming^50,51^ and reflects previous work linking respiration to temperature in aquatic systems^50,52,53^. Whole-system warming experiments highlight the role of temperature in increasing respiration relative to production, reducing carbon sequestration^54^.

How system-general are the relationships we observed? The differences between nighttime and daytime respiration rates were highly seasonal, occurring in the spring and early summer, when productivity (enhancing O_2_ levels^6^) and solar insolation (enhancing temperature^55^) were highest (Fig. 1b). Community respiration rates increased as both temperature and O_2_ levels increased (Fig. 2), reflecting higher temperatures and dissolved O_2_ concentrations during the day (Fig. 3). Thus, the assumption that nighttime and daytime respiration rates are comparable^22^ is likely to be violated in locations where there is substantial seasonal and diel variation in environmental factors (e.g., temperature, O_2_) that influence respiration. Thus, smaller and shallower bodies of water (*e.g.*, lakes, lagoons, estuaries, tide pools) are more likely to exhibit variation than larger ones^35,37,56,57^.

In conclusion, we found that measuring whole-community respiration rates during day and night—which was perhaps uniquely possible in the contained water of tide pools—resulted in markedly different estimates of respiration and production. These differences were associated with substantial variation in environmental conditions during night and day. Especially in systems characterized by diel variation in factors such as temperature and O_2_, the assumption that daytime and nighttime respiration rates are equivalent may not hold^57^. In these cases, if in-situ daytime respiration rates cannot be accurately measured, there may be a need to follow an alternative method for estimating gross community production and other key components of ecosystem metabolism (*e.g.*, the triple oxygen tracer approach^57,58^) or to incorporate modeling approaches that relate ecosystem metabolism to temperature^59^ and O_2_. More broadly, our work demonstrates that temperature and O_2_ concentrations—both of which are changing due to human activities^1,6^—can strongly affect rates of ecosystem metabolism, influencing system- and global-scale processes such as carbon sequestration.

## Methods

### Study site and measurement schedule

Our study was conducted on rocky intertidal reefs at John Brown’s Beach on Japonski Island, Sitka, Alaska, USA (57.06° N, 135.37° W)^60^ between September 2018 and September 2019. Measurements spanned a year to capture effects of season and temperature on ecosystem metabolism^61^. Tide pools (*n* = 5, randomly selected from pools at the site) ranged from 3 to 28 L in volume, averaging 9.4 ± 4.7 L (mean ± s.e.m.), and were located from 2.3 to 3.0 m in elevation above mean lower-low water, averaging 2.7 ± 0.1 m. Pool water temperatures ranged from a minimum of 4.6 °C in January to a maximum of 23.1 °C in July. Daylight hours (sunrise-to-sunset) during measurements ranged from 7.5 h in January to 17.9 h in June. Irradiance levels during sampling events ranged from 62 to 232 µmol photons m^−2^ s^−1^. Sampling occurred across a > 1 year period on the following dates: 3–12 Sep 2018; 16–21 Jan 2019; 26–31 Mar 2019; 28 Apr–2 May 2019; 11–14 Jun 2019; 7–11 Jul 2019; 5–7 Aug 2019; and 19–22 Sep 2019.

### Measurements taken

During each sampling event, we collected two complementary sets of data to evaluate changes in O_2_ concentrations in the pools. All data from the study are available in the *Dryad* repository, 10.7280/D1M39B^62^. The first dataset consisted of daytime and nighttime (Day–Night) sampling, where pools were left uncovered and light varied naturally on a diel cycle. During one daytime and one nighttime low tide event, we took in-situ measurements and collected water samples. We took initial readings of O_2_ (mg L^−1^; DSS optical DO meter, YSI, Inc., Yellow Springs, Ohio, USA), temperature (°C; YSI DSS), and light (photosynthetically active radiation in µmol photons m^−2^ s^−1^; MQ 210 Underwater Quantum Meter, Apogee Instruments, Logan, Utah, USA) in the water column of each pool as soon as the pool was isolated by the receding tide. After a minimum of 1 h (median = 1.5 h, max = 3.5 h), pools were resampled, and a second set of measurements were taken. Daytime measurements were taken on days when irradiance levels were high enough to saturate photosynthetic rates of the most abundant seaweed species in the tide pools, *Neorhodomela oregona* (> 47 µmol photons m^−2^ s^−1^) based on light curves measured using pulse amplitude modulated fluorometry (M. Bracken, unpublished data). Nighttime samples were collected on nights when the pools were isolated by the tide after dark (measured irradiance levels of 0.0 µmol photons m^−2^ s^−1^).

The second set of measurements consisted of light and dark (Light–Dark) sampling during the daytime. Protocols were similar to those used to calculate light–dark bottle estimates of productivity in oceanographic and limnological applications^12,20^, modified for in-situ measurements of oxygen fluxes in tide pools to estimate net community production, community respiration, and gross community production^19,39^. Measurements were started shortly after pools were isolated by the receding tide, when irradiance levels were sufficient to saturate photosynthesis by *N. oregona* (see above). Immediately after initial measurements of O_2_, irradiance, and temperature (see above), pools were covered with opaque black plastic sheeting (6-mil black polyurethane plastic sheeting, Film Gard, Berry Plastics, Evansville, Indiana, USA), which was anchored to the substratum with cobbles to ensure that no light entered the pool during the “Dark” incubation. “Dark” incubations were conducted prior to “Light” incubations to minimize the potential for high initial O_2_ concentrations, which could lead to photorespiration^63^ and reduce the concentration gradient between the primary producers and the tide pool water. Time of day did not affect initial dissolved O_2_ concentrations (*F*_1,23_ = 1.3, *p* = 0.259), likely because measurements were made as soon as possible after the receding tide isolated the tide pools. Irradiance levels beneath the plastic sheeting were uniformly 0.0 µmol photons m^−2^ s^−1^, and the sheeting did not alter tide pool temperatures during the “Dark” incubation (mean ± s.e.m. = − 0.04 ± 0.16 °C; *t* = 0.2*, d.f.* = 4, *p* = 0.832). After an incubation period of at least 30 min (median = 45 min, maximum = 80 min), we made a second set of measurements and removed the plastic sheeting, initiating a “Light” incubation of at least 30 min. At the end of the “Light” incubation, a final set of measurements was made.

We made additional measurements to ensure that covering tide pools did not affect rates of production and respiration. During June and July, when differences between daytime and nighttime conditions were likely to be greatest, we measured daytime changes in O_2_ concentrations with and without transparent plastic sheeting (8-mil Crystal Clear vinyl sheeting, Frost King, Thermwell Products Co., Inc., Matwah, New Jersey, USA) and found that rates of O_2_ accumulation were not different in transparent-covered and uncovered pools (paired *t*-test: *t* = 0.5, *d.f.* = 4, *p* = 0.761; Supplementary Fig. S4). Light levels did not differ between uncovered and transparent-covered pools (paired *t*-test: *t* = 2.2, *d.f.* = 4, *p* = 0.248). Note that some UV radiation was likely blocked by the vinyl sheeting, but the lack of a difference in O_2_ accumulation rates suggests that effects were minimal. Similarly, we conducted nighttime incubations with and without opaque plastic sheeting and found that rates of O_2_ depletion were not different in opaque-covered and uncovered pools (paired *t*-test: *t* = 1.2, *d.f.* = 4, *p* = 0.505; Supplementary Fig. S4). Furthermore, median wind velocities (measured at the adjacent Rocky Gutierrez Airport) were low enough during sampling (median = 2.8 m s^−1^) that O_2_ diffusion across the air–water interface should not have affected measurements^19^, and thus covering tide pools at night should not have been necessary. Wind speeds during measurements were typical of velocities measured throughout the year (median = 2.6 m s^−1^).

### Analyses

We measured changes in O_2_ concentrations in the light (Light and Day) to estimate net community production (*NCP*) and changes in O_2_ concentrations in the dark (Dark and Night) to estimate community respiration (*CR*). In both cases, we calculated the difference between the final (*f*) and initial (*i*) concentrations and divided by the elapsed time (*t*) in hours:

1 $$NCP= \frac{({{O}_{2}<light>}_{f} - {{O}_{2}<light>}_{i})}{({t}_{f}- {t}_{i})}$$

2 $$CR= \frac{({{O}_{2}<dark>}_{f} - {{O}_{2}<dark>}_{i})}{({t}_{f}- {t}_{i})}$$

Values were calculated in mg O_2_ L^−1^ h^−1^, as those units reflected the concentrations measured on our optical DO meter. However, tide pool volume and area were strongly correlated (*r*^2^ = 0.97), we used estimates of volume (L) and area (m^2^) to calculate O_2_ changes in mg m^−2^ h^−1^, and we include those values in the Supplementary Information (Supplementary Fig. S1). Estimates of *NCP* were typically positive, due to increases in O_2_ concentrations in the light, whereas estimates of *CR* were typically negative, due to declines in O_2_ concentrations in the dark. Independent estimates of *NCP* and *CR* were made using each set of measurements: Day–Night (daytime *NCP* and nighttime *CR*) and Light–Dark (Light *NCP* and Dark *CR*).

Gross community production (*GCP*) was estimated by adding together net community production (*NCP*) and the absolute value of community respiration (*CR*):

3 $$GCP= NCP+ \left|CR\right|$$

Independent estimates of *GCP* were made using the Day–Night (daytime *NCP* + nighttime *CR*) measurements and the Light–Dark (light *NCP* + dark *CR*) measurements.

To evaluate differences between methods, we used repeated-measures analyses of variance in SAS v. 9.4 (SAS Institute, Inc., Cary, North Carolina, USA), after verifying both univariate and multivariate assumptions of normality and homogeneity of variances. Analyses were conducted using *proc mixed*, with ‘Date’ as the within-subjects variable (i.e., measurements were repeated at each pool over time) and ‘Pool’ designated as the subject. Comparisons were made between methods (e.g., oxygen fluxes measured in daytime “Dark” incubations vs. oxygen fluxes measured at “Night”), including changes through time (i.e., ‘Method × Date’ interactions). We also explored the role of environmental factors (temperature, O_2_, light) in driving variation in productivity using the same analytical structure.

### Daily and annual productivity estimates

We generated estimates of 24-h, integrated community respiration (*CR*) and net community production (*NCP*), and daylight gross community production (*GCP*) for a 1-yr period from 18 Sep 2018 to 17 Sep 2019 that fell within the range of our sampling dates. We made the simplifying assumption that O_2_ fluxes would be the same in submerged pools as in isolated pools. This is a reasonable assumption, as these pools are high on the shore (2.3 to 3.0 m above mean lower-low water) relative to the maximum high water level during the year (3.8 m) and, based on tidal predictions, were submerged for only 10.5 ± 3.2% of the time over the year. We also made the assumption that for the primary producers in the tide pools, irradiance would be saturating immediately at sunrise and until sunset, without attempting to account for individual patterns of incident irradiance in each pool. This assumption is reasonable given the low irradiance levels (< 50 µmol photons m^−2^ s^−1^) required to saturate photosynthesis of the most abundant seaweed species in the tide pools (see above). Using the values measured in the pools on the 8 sampling dates, we linearly interpolated values of *CR*, *NCP*, and *GCP* for both the Light–Dark and Day–Night determination methods, for each intervening day.

For each day in the 365 d dataset, we calculated daily *CR* (mg O_2_ L^−1^ d^−1^) using *CR* estimates from the (1) daytime “Dark” measurements only, (2) Night measurements only, and (3) Dark measurements during daylight hours and Night measurements during nighttime hours. Similarly, we used three methods to calculate integrated estimates of daily *NCP* (mg O_2_ L^−1^ d^−1^) over each 24 h period. (1) For the Light–Dark method, we used the “Light” *NCP* measurement multiplied by the number of daylight hours, and then subtracted the “Dark” *CR* under the opaque plastic sheet, multiplied by the number of nighttime hours to estimate the total 24 h *NCP*. (2) For the Day–Night method we used the “Day” *NCP* estimate multiplied by the number of daylight hours, and the “Night” *CR* value multiplied by the number of nighttime hours to calculate a 24 h *NCP* value. (3) We also combined the two methods, using the Light–Dark daytime (“Light”) *NCP* estimate multiplied by the number of daylight hours, and the Day–Night nighttime (“Night”) *CR* estimate multiplied by the number of nighttime hours to calculate an integrated 24 h value.

Integrated estimates of gross community production (*GCP*, mg O_2_ L^−1^ d^−1^)—which only occurs during daylight hours when oxygen is produced and consumed—were calculated from the *NCP* value plus the *CR* value for the Light–Dark and Day–Night methods, multiplied by the number of daylight hours on each date.

## Supplementary Information

Supplementary Information.

## Data Availability

The datasets generated during and/or analyzed during the current study are available in the Dryad repository, 10.7280/D1M39B^62^.
