# Supplementary material for: Accounting for variation in temperature and oxygen availability when quantifying marine ecosystem metabolism
Source: Sci Rep. 2022 Jan 17;12:825. doi: 10.1038/s41598-021-04685-8 (PMC8763951; doi:10.1038/s41598-021-04685-8)
Supplement: Supplementary file 1 — Supplementary Information. [file 41598_2021_4685_MOESM1_ESM.pdf]

Supplementary Information for

Accounting for variation in temperature and oxygen availability when quantifying marine ecosystem metabolism

Matthew E. S. Bracken, Luke P. Miller, Sarah E. Mastroni, Stephany M. Lira, and Cascade J. B. Sorte

Correspondence: m.bracken@uci.edu

ORCID iDs: M.E.S.B. https://orcid.org/0000-0002-0068-7485; L.P.M. https://orcid.org/0000-0002-2009-6981; C.J.B.S. https://orcid.org/0000-0003-0952-951X

This PDF file includes Supplementary Figures S1-S4.

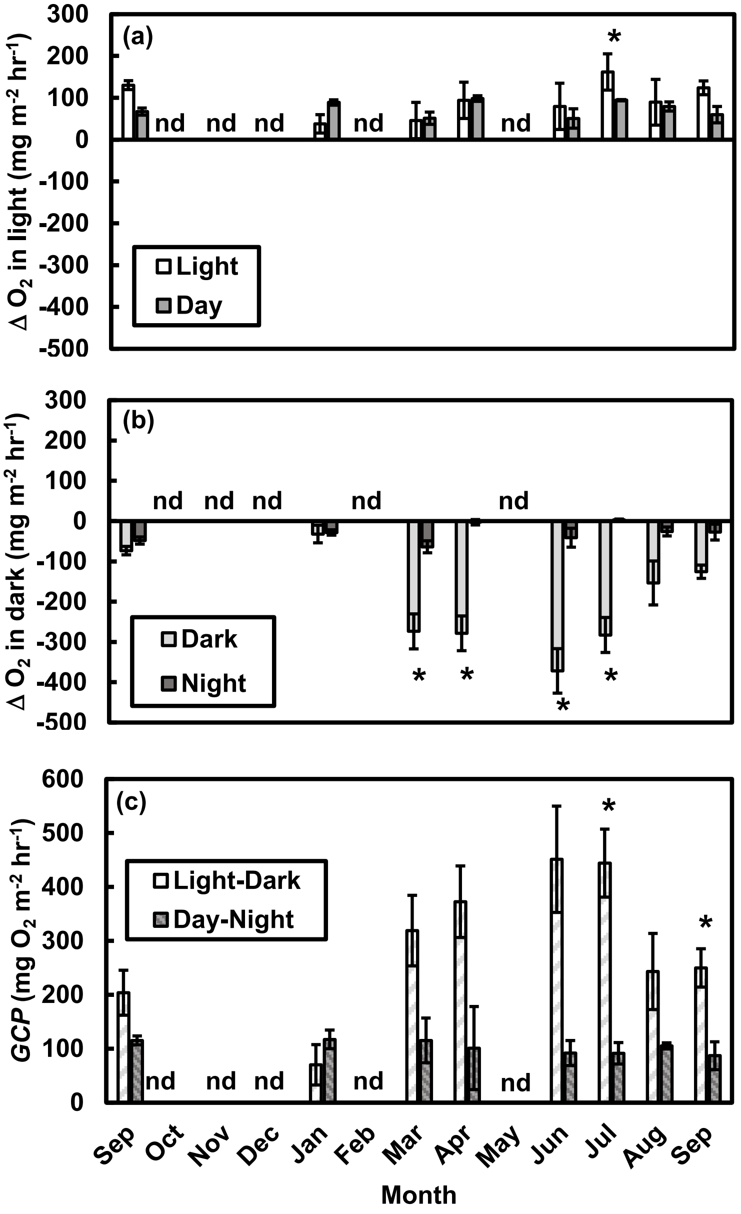

Fig. S1 Effects of methodology on measured changes in oxygen (O_2) concentrations and gross community production (*GCP*) in tide pools, expressed on a per-area basis. **(a)** Measurements made in the light (estimates of net community production) generally indicated net production of O_2, were similar regardless of method ("Light" vs. "Day"), and did not change over time. **(b)** Measurements in the dark (estimates of community respiration) typically indicated net consumption of O_2, differed substantially depending on method ("Dark" vs. "Night"), and changed over time. **(c)** Measurements of *GCP* made consecutively during daytime (Light-Dark) resulted in higher estimates of *GCP* than those made during day and night (Day-Night). Overall, *GCP* varied with time, and the difference between methods changed with time. Values are means ± s.e., and "nd" marks months when "no data" were collected. Asterisks (*) indicate differences between methods ($p < 0.05$).

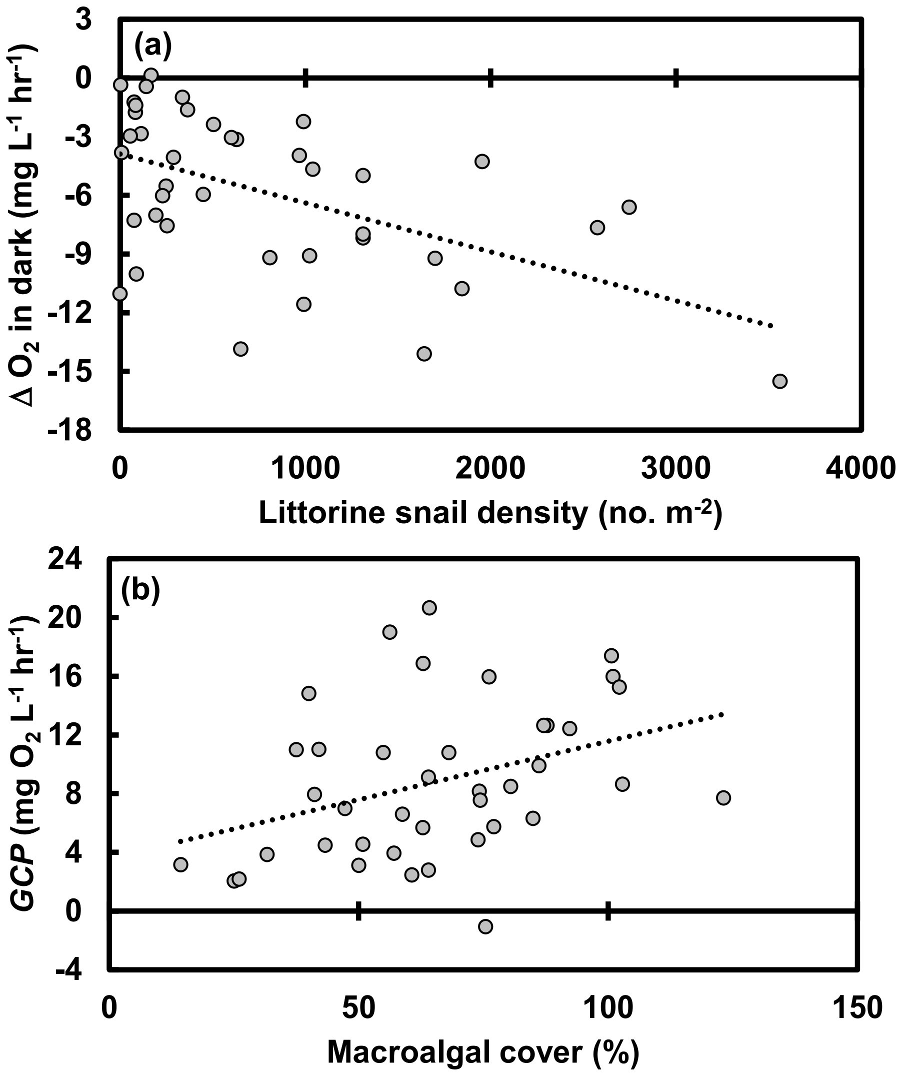

Fig. S2. Organisms associated with respiration and production in tide pools. (a) Community respiration rates (*CR*; depletion of O_2 during the daytime in darkened "Dark" tide pools) were greater in tide pools containing higher densities of littorine snails ($p < 0.001$). (b) Gross community production (*GCP*) was higher in tide pools with higher macroalgal cover ($p = 0.029$). Note that algal cover could exceed 100% due to layering of algal canopy over understory species.

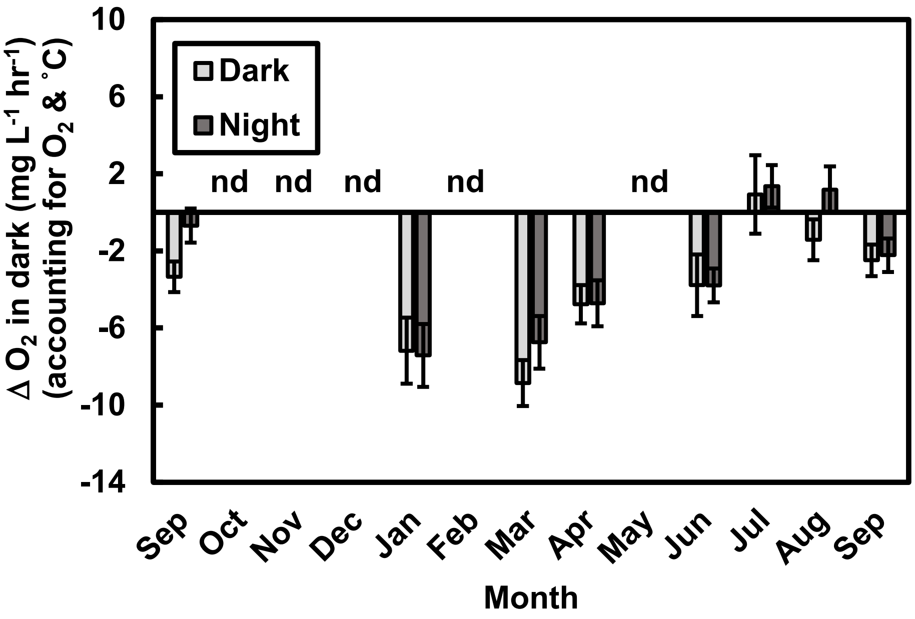

Fig. S3. Effects of methodology on measured changes in oxygen (O_2) in the dark after accounting for initial temperatures and O_2 concentrations. Measurements typically indicated net consumption of O_2 (i.e., most values were negative) and changed over time, but they did not differ depending on method. Values are least-squares means (after accounting for O_2 and temperature) ± standard errors, and "nd" refers to "not determined".

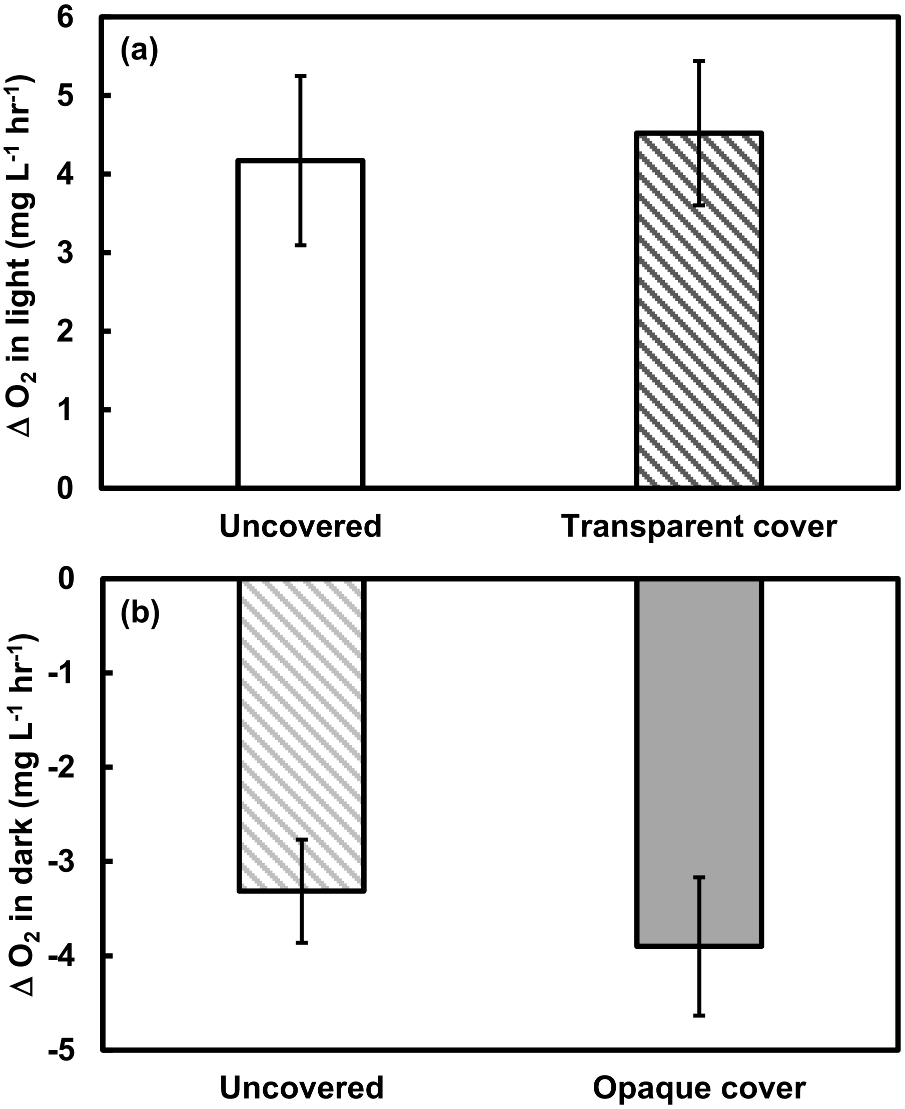

Fig. S4. Effects of plastic covers on changes in oxygen (O_2) concentrations in the (a) light and (b) dark. Measurements in the light were conducted during the day, when light intensities were > 225 μmol photons m^{-2} s^{-1}. Measurements in the dark were conducted at night, when light levels were below the detection limit of our light intensity meter. Covering tide pools with plastic sheeting had no effect on changes in oxygen concentrations ($p > 0.50$ in both cases). Values are means ± standard errors.
